# Supplementary material for: Impact of the Covid-19 pandemic on audiology service delivery: Observational study of the role of social media in patient communication
Source: PLoS One. 2024 Apr 25;19(4):e0288223. doi: 10.1371/journal.pone.0288223 (PMC11045075; doi:10.1371/journal.pone.0288223)
Supplement: Attachment — Submitted filename: PONE-D-23-19309_reviewer.pdf [file pone.0288223.s001.pdf]

PLOS ONE

Impact of the Covid-19 Pandemic on Audiology Service Delivery: Observational Study of the Role of Social Media in Patient Communication

--Manuscript Draft--

**Manuscript Number:**	PONE-D-23-19309
**Article Type:**	Research Article
**Full Title:**	Impact of the Covid-19 Pandemic on Audiology Service Delivery: Observational Study of the Role of Social Media in Patient Communication
**Short Title:**	Impact of the Covid-19 Pandemic on Audiology Service Delivery
**Corresponding Author:**	Zain Hussain<br>The University of Edinburgh<br>Edinburgh, UNITED KINGDOM
**Keywords:**	Covid-19, Data mining, Audiology, Service change
**Abstract:**	The Covid-19 pandemic has highlighted an era in hearing health care that necessitates a comprehensive rethinking of audiology service delivery. There has been a significant increase in the number of individuals with hearing loss who seek information online. An estimated 430 million individuals worldwide suffer from hearing loss, including 11million in the United Kingdom. The objective of this study was to identify NHS audiology service social media posts and understand how they were used to communicate service changes within audiology departments at the onset of the Covid-19 pandemic.Facebook and Twitter posts relating to audiology were extracted over a six week period (March 23 to April 30 2020) from the United Kingdom. We manually filtered the posts to remove those not directly linked to NHS audiology service communication. The extracted data was then geospatially mapped, and themes of interest were identified via a manual review. We also calculated interactions (likes, shares, comments) per post to determine the posts' efficacy. A total of 981 Facebook and 291 Twitter posts were initially mined using our keywords, and following filtration, 174 posts related to NHS audiology change of service were included for analysis. The results were then analysed geographically, along with an assessment of the interactions within the included posts. NHS Trusts and Boards should consider incorporating and promoting social media to communicate service changes. Users would be notified of service modifications in real-time, and different modalities could be used (e.g. videos), resulting in a more efficient service.
**Order of Authors:**	Adeel Hussain
	Zain Hussain
	Mandar Gogate
	Kia Dashtipour
	Adele Goman
	Aziz Sheikh
	Amir Hussain
**Additional Information:**	
**Question**	**Response**
**Financial Disclosure**<br><br>Enter a financial disclosure statement that describes the sources of funding for the work included in this submission. Review the submission guidelines for detailed requirements. View published research articles from *PLOS ONE* for specific	The author(s) received no specific funding for this work.

examples.<br><br>This statement is required for submission and **will appear in the published article** if the submission is accepted. Please make sure it is accurate.<br><br>**Unfunded studies**<br>Enter: *The author(s) received no specific funding for this work.*<br><br>**Funded studies**<br>Enter a statement with the following details:<br>• Initials of the authors who received each award<br>• Grant numbers awarded to each author<br>• The full name of each funder<br>• URL of each funder website<br>• Did the sponsors or funders play any role in the study design, data collection and analysis, decision to publish, or preparation of the manuscript?<br>• **NO** - Include this sentence at the end of your statement: *The funders had no role in study design, data collection and analysis, decision to publish, or preparation of the manuscript.*<br>• **YES** - Specify the role(s) played.<br><br>* typeset	
**Competing Interests**<br><br>Use the instructions below to enter a competing interest statement for this submission. On behalf of all authors, disclose any competing interests that could be perceived to bias this work—acknowledging all financial support and any other relevant financial or non-financial competing interests.<br><br>This statement is required for submission and **will appear in the published article** if the submission is accepted. Please make sure it is accurate and that any funding sources listed in your Funding Information later in the submission form are also declared in your Financial Disclosure statement.	The authors have declared that no competing interests exist.

View published research articles from *PLOS ONE* for specific examples.<br><br>**NO authors have competing interests**<br><br>Enter: *The authors have declared that no competing interests exist.*<br><br>**Authors with competing interests**<br><br>Enter competing interest details beginning with this statement:<br><br>*I have read the journal's policy and the authors of this manuscript have the following competing interests: [insert competing interests here]*<br><br>* typeset	
**Ethics Statement**<br><br>Enter an ethics statement for this submission. This statement is required if the study involved:<br><br>• Human participants<br>• Human specimens or tissue<br>• Vertebrate animals or cephalopods<br>• Vertebrate embryos or tissues<br>• Field research<br><br>Write "N/A" if the submission does not require an ethics statement.<br><br>General guidance is provided below. Consult the submission guidelines for detailed instructions. **Make sure that all information entered here is included in the Methods section of the manuscript.**	N/A

**Format for specific study types**<br><br>**Human Subject Research (involving human participants and/or tissue)**<br>• Give the name of the institutional review board or ethics committee that approved the study<br>• Include the approval number and/or a statement indicating approval of this research<br>• Indicate the form of consent obtained (written/oral) or the reason that consent was not obtained (e.g. the data were analyzed anonymously)<br><br>**Animal Research (involving vertebrate animals, embryos or tissues)**<br>• Provide the name of the Institutional Animal Care and Use Committee (IACUC) or other relevant ethics board that reviewed the study protocol, and indicate whether they approved this research or granted a formal waiver of ethical approval<br>• Include an approval number if one was obtained<br>• If the study involved *non-human primates*, add *additional details* about animal welfare and steps taken to ameliorate suffering<br>• If anesthesia, euthanasia, or any kind of animal sacrifice is part of the study, include briefly which substances and/or methods were applied<br><br>**Field Research**<br><br>Include the following details if this study involves the collection of plant, animal, or other materials from a natural setting:<br>• Field permit number<br>• Name of the institution or relevant body that granted permission	
**Data Availability**<br><br>Authors are required to make all data underlying the findings described fully available, without restriction, and from the time of publication. PLOS allows rare exceptions to address legal and ethical concerns. See the PLOS Data Policy and FAQ for detailed information.	Yes - all data are fully available without restriction

A Data Availability Statement describing where the data can be found is required at submission. Your answers to this question constitute the Data Availability Statement and **will be published in the article**, if accepted.<br><br>**Important:** Stating 'data available on request from the author' is not sufficient. If your data are only available upon request, select 'No' for the first question and explain your exceptional situation in the text box.<br><br>Do the authors confirm that all data underlying the findings described in their manuscript are fully available without restriction?	
**Describe where the data may be found in full sentences. If you are copying our sample text, replace any instances of XXX with the appropriate details.**<br><br>• If the data are **held or will be held in a public repository**, include URLs, accession numbers or DOIs. If this information will only be available after acceptance, indicate this by ticking the box below. For example: *All XXX files are available from the XXX database (accession number(s) XXX, XXX.).*<br>• If the data are all contained **within the manuscript and/or Supporting Information files**, enter the following: *All relevant data are within the manuscript and its Supporting Information files.*<br>• If neither of these applies but you are able to provide **details of access elsewhere**, with or without limitations, please do so. For example:<br><br>*Data cannot be shared publicly because of [XXX]. Data are available from the XXX Institutional Data Access / Ethics Committee (contact via XXX) for researchers who meet the criteria for access to confidential data.*<br><br>*The data underlying the results presented in the study are available from (include the name of the third party*	All relevant data are within the manuscript and its Supporting Information files.

and contact information or URL).<br>• This text is appropriate if the data are owned by a third party and authors do not have permission to share the data.<br><br>* typeset	
Additional data availability information:	

Manuscript

Click here to access/download;Manuscript;LaTeX_Template_for_PLOS__Publ

Impact of the Covid-19 Pandemic on Audiology Service Delivery: Observational Study of the Role of Social Media in Patient Communication

Adeel Hussain[1], Zain Hussain[2*], Mandar Gogate[1], Kia Dashtipour[1], Adele Goman[1], Aziz Sheikh[3], Amir Hussain[1],

1 School of Computing, Edinburgh Napier University, Edinburgh, EH10 5DT, Scotland
2 The University of Edinburgh, Chancellor's Building, 49 Little France Crescent, Edinburgh, EH16 4SB
3 Usher Institute,The University of Edinburgh, Old Medical School, Teviot Place, Edinburgh, EH8 9AGA, Scotland

* zain.hussain@ed.ac.uk

Abstract

The Covid-19 pandemic has highlighted an era in hearing health care that necessitates a comprehensive rethinking of audiology service delivery. There has been a significant increase in the number of individuals with hearing loss who seek information online. An estimated 430 million individuals worldwide suffer from hearing loss, including 11 million in the United Kingdom. The objective of this study was to identify NHS audiology service social media posts and understand how they were used to communicate service changes within audiology departments at the onset of the Covid-19 pandemic.Facebook and Twitter posts relating to audiology were extracted over a six week period (March 23 to April 30 2020) from the United Kingdom. We manually filtered the posts to remove those not directly linked to NHS audiology service communication. The extracted data was then geospatially mapped, and themes of interest were identified via a manual review. We also calculated interactions (likes, shares, comments) per post to determine the posts' efficacy. A total of 981 Facebook and 291 Twitter posts were initially mined using our keywords, and following filtration, 174 posts related to NHS audiology change of service were included for analysis. The results were then analysed geographically, along with an assessment of the interactions within the included posts. NHS Trusts and Boards should consider incorporating and promoting social media to communicate service changes. Users would be notified of service modifications in real-time, and different modalities could be used (e.g. videos), resulting in a more efficient service.

Introduction

To mitigate the exponential spread of Covid-19 cases, the government took quick and decisive steps that affected the healthcare services, including audiology departments in the UK. Consequently, there was a transition from in-person to teleaudiology patient care, which resulted in the cancellation of all in-person appointments for routine services. Moreover, new assessments and existing hearing aid users were no longer able to access their regular care pathways in the event of hearing difficulties [1, 2].

Prior to the service being shifted to remote or postal services, audiology was predominantly a face-to-face facility for patients. However, due to the lack of

teleaudiology infrastructure, certain procedures, such as otoscopy, the necessity for sound-treated rooms for testing, and multiple face-to-face appointments for hearing aid fitting, counselling, and troubleshooting could not be performed remotely. Previous research [2, 3] has shown some reticence towards teleaudiology being used in clinical settings. Eikelboom and Swanepoel [3], surveyed 269 audiologists from around the world and found that only 15% had used teleaudiology, despite being confident in using the required technology [3]. Similarly, Saunders and Roughley surveyed 120 UK-based audiologists during the Covid-19 pandemic and found that while 98% of participants used teleaudiology at the time of the study, only 30% had used it beforehand. Participants responded positively to the remote pathways, but also highlighted the need for improvements in training and infrastructure [4]. In addition, the lack of infrastructure and the inability to perform certain tasks, teleaudiology services had to be utilised during national and regional lockdowns. In recent decades, patients are increasingly seeking more information, making informed rehabilitation decisions, and taking responsibility for managing their conditions. Previously, individuals with a disability or health issue would seek advice and assistance from the internet, their friends, family, or healthcare experts. With improved internet accessibility and social media, individuals have more autonomy in gathering information from a wide range of resources [5]. During the Covid-19 pandemic, face-to-face communication with healthcare providers decreased, which has exacerbated this trend. In April 2020, research studies showed that people in the United Kingdom spent an average of four hours a day online, up from three hours and 29 minutes in September 2019, further supporting the aforementioned [6]. A recent study by Gupta ~~et al~~ [7] found Facebook and Twitter to be the most commonly used type of social media. Approximately 430 million people worldwide and 11 million individuals in the United Kingdom are estimated to have some degree of hearing loss, approximately, 1 in 6 around 900,000 of them having severe or profound hearing loss [8]. A listener's level of difficulty will vary based on the type of hearing loss they have, the environment they are in, whether their hearing loss has been treated, and how effectively their hearing aids are functioning. Since face-to-face communication was restricted due to the lockdown, a written source of information would have been beneficial if a listener had non-functioning hearing aids who were unable to participate in a telephone discussion. Our study is the first of its kind to investigate the role and impact of social media in engaging audiology consumers on service changes during the initial 6 weeks of the Covid-19 pandemic. Specifically, we aim to analyse how audiology departments across health trusts in the United Kingdom utilised Facebook and Twitter, to communicate with their patients.

Materials and methods

Ethics

Due to the availability of the data used in this study in the public domain, no NHS ethics review was required. Prior to previous research and to ensure compliance with pertinent provisions of the General Data Protection Regulation (GDPR) [9, 10], a comprehensive assessment was conducted to confirm that our study presented no privacy risk to individuals. We aimed to follow best practices for user privacy by excluding private information from our dataset. In addition, to comply with privacy laws and social media policies in accordance with the GDPR, to collect data, we did not disclose or published direct posts by individuals, quotations from individuals, or the names or locations of users who are not public organisations or entities on the Facebook CrowdTangle platform and Twitter API [11].

Data sources

We opted to use data from Facebook and Twitter, since they are the most used social media platforms [7]. Within the period from March 23 to April 30, 2020, we specifically targeted English-language Facebook posts from pages in the UK. This particular time frame was selected due to its alignment with the initial announcement of the first UK lockdown [9]. In order to identify which audiology departments were utilising social media to communicate service changes, we narrowed our search to only include the initial six-week period of the lockdown. Furthermore, to collect Facebook posts and tweets, we employed the Crowdtangle service and Twitter API [12].

The search terms used to extract data were hearing loss, hearing, difficulty, presbycusis, tinnitus, deafness, speech impairment, hearing aids, audiology, ear wax, ear syringing, microsuction, telecare, teleaudiology remote consultations. The comprehensive range of our search terms ensured that all relevant results were included during the searches. Our initial search yielded 981 Facebook posts and 291 Twitter posts, which were then filtered to exclude posts unrelated to the NHS audiology departments. Our team manually assessed each post for messages of service change from NHS Trusts or partners, such as libraries where repair/battery clinics had previously been held. The final dataset did not include posts about private hearing clinics, other hearing-related news, or information from news outlets. This study also omitted information from the private sector and hearing aid manufacturers.

Data analysis

Crowd tangle and Twitter API was used to calculate the frequencies of the various interactions from the filtered dataset. For parameters of interest, frequencies were calculated, and circular charts, tables, and heat maps illustrated the results. The presented heat map depicts the diverse geographical origins of the posts, with the colour and size of the circles serving as indicators of the quantity of interactions.

Results and Discussion

In this study, we investigated how NHS audiology departments utilised social media to provide service updates to patients. Fig 1illustrates that most posts were generated between the third and the fifth weeks.

Table 1 shows that out of 14 NHS Boards in Scotland, seven reported changes in service information on Facebook. Similarly, out of 217 NHS trusts in England, 41 reported changes in service information on Facebook and Twitter. In Wales out of 7 trusts, three reported a change in service information, and in Northern Ireland, out of five trusts only one reported a change in service information.

There was a total of 3,646 interactions across 174 posts, Table 2 illustrates the responses and interactions from the different countries across the UK. To be noted, this sample represents only a fraction of 11 million hearing aid users in the UK [8]. Interactions included likes, shares, comments, or reposts. However, it's possible that some people who viewed the posts did not interact with them.

Fig 2 shows a visual representation of the interactions. Similarly, the bubble map indicates that the areas with a higher number of responses corresponded to areas with a higher number of interactions. In addition, the number of responses from the areas that posted service change updates in the U.K. Both the gradient of the filled circle and the size of the circle indicate the number of posts and the number of interactions per post using the Virdis scale (blue-¿yellow and small-¿large indicating an increasing number of interactions

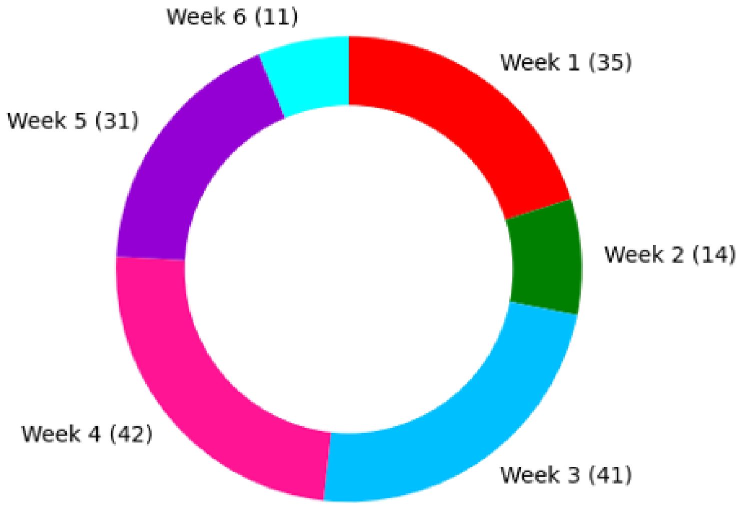

Fig 1. Number of weekly posts

Table 1. Number of Trusts and Boards in different parts of the United Kingdom.

**Area**	**Number of NHS trusts boards**	**Number of trusts boards with posts of service change**
England	217	41
Scotland	14	7
Wales	7	3
Northern Ireland	5	1

It has been shown that individuals who have hearing loss can benefit from utilising electronic media as it improves their communication abilities and reduces auditory barriers [13]. Therefore, internet usage appeals to those who prefer text-based communication [14]. It is recommended to leverage the impact of social media for obtaining service change updates, given that a study by Manchaiah et al [5] found that Facebook and YouTube were the most frequently used social media platforms, with over 40% of participants reporting using them in their study [5]. The current study provides evidence that sharing service change messages on reliable online platforms can effectively inform patients. However, the credibility of information shared on social media can be undermined by the circulation of false or misleading content, as indicated by previous research [7]. The failure to timely post accurate information on social media may result from the ambiguity surrounding lockdown protocols and a shortage of personnel due to the government furlough programme, which was initiated on 27 March 2020.

Table 2. Number of shares, likes and comments on posts in England, Scotland, Wales and N. Ireland

Country	Likes	Shares	Comments	CTotal
England	915	856	236	2007
Scotland	415	737	323	1475
Wales	84	161	29	274
Northern Ireland	66	14	4	84

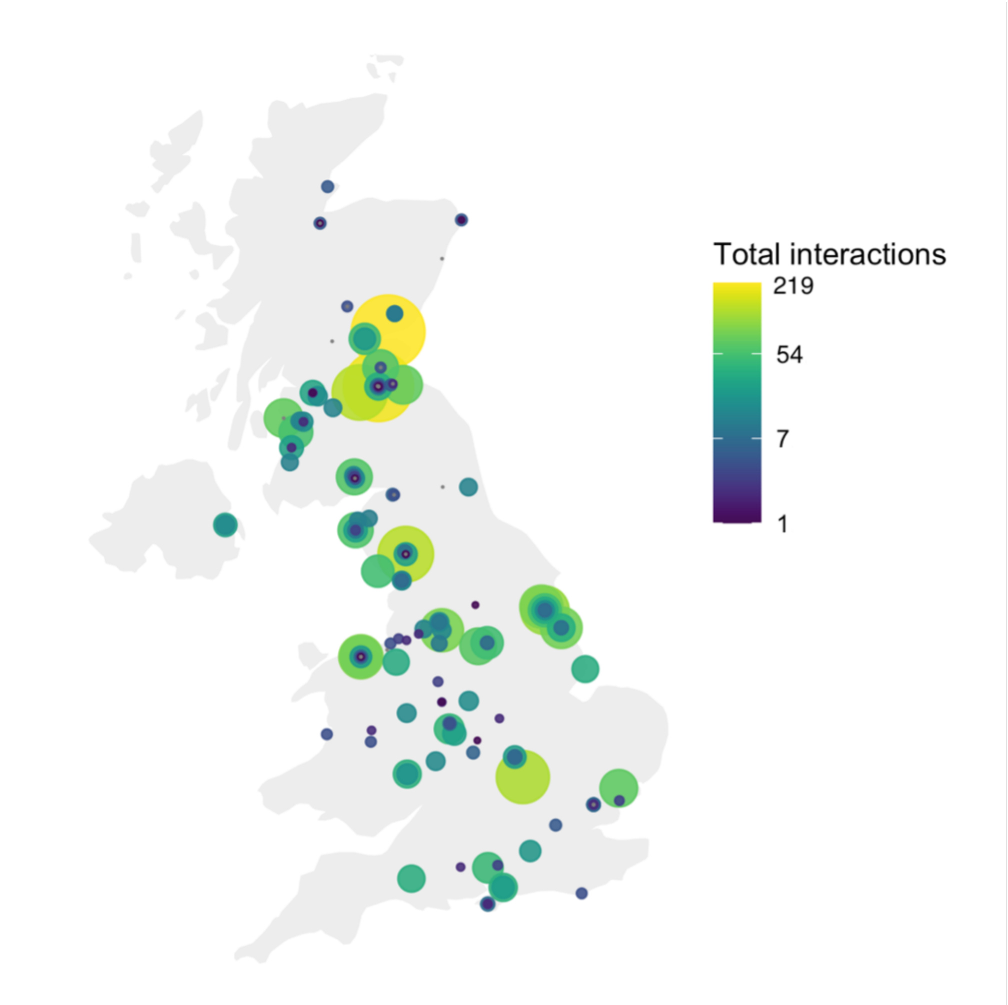

Fig 2
The number of responses from the areas that posted service change updates in the U.K.

Strengths and limitations

This study demonstrated its strengths by extracting data from user-frequented sites that were appropriate for the research. However, due to the data protection laws of the NHS, we were unable to access information on emails, SMS, or letters addressed that were sent to patients regarding changes in the service.

Future directions

This research is the first to evaluate social media sites to investigate notifications of changes to the audiology service. In future research, we will further extend this study by incorporating additional platforms, including other electronic media sites such as, TikTok and YouTube. To enhance our understanding of the communication strategies employed by healthcare providers, we may also consider supplementing social media-mined data with surveys sent to various National Health Service (NHS) trusts. Such surveys will help us to determine the approaches utilised by these trusts in informing patients of service changes and suggest potential areas for improvement to deliver a superior service.

Conclusion

The current study investigated how several NHS audiology departments used social media to notify patients of service changes. It found that social media was not being used to its maximum potential by many trusts in the UK, as supported by the lack of posts or information on service changes on their Facebook sites and even less on Twitter. Audiology healthcare practitioners who work with patients with hearing loss, should be aware of how crucial information accessible via electronic media facilitates service accessibility for individuals with hearing loss. A well-informed workforce would provide more effective service and would in turn lead to fewer patient complaints. Social media has been utilised in the past for information dissemination and updates during public health crises [15].

Acknowledgments

This research is supported by the UK EPSRC COG-MHEAR programme (Grant No. EP/M026981/1).

References

1. Swanepoel DW, Hall JW. Making audiology work during COVID-19 and beyond. The Hearing Journal. 2020;73(6):20–22.
2. Parmar B, Beukes E, Rajasingam S. The impact of COVID-19 on provision of UK audiology services & on attitudes towards delivery of telehealth services. International Journal of Audiology. 2022;61(3):228–238.
3. Eikelboom RH, Swanepoel DW. International survey of audiologists' attitudes toward telehealth. American Journal of Audiology. 2016;25(3S):295–298.
4. Saunders GH, Roughley A. Audiology in the time of COVID-19: practices and opinions of audiologists in the UK. International Journal of Audiology. 2021;60(4):255–262.
5. Manchaiah V, Bellon-Harn ML, Kelly-Campbell RJ, Beukes EW, Bailey A, Pyykkő I. Media use by older adults with hearing loss: An exploratory survey. American Journal of Audiology. 2020;29(2):218–225.
6. UK's internet use surges to record levels; 2020. https://www.ofcom.org.uk/news-centre/2020/uk-internet-use-surges#:~:text=The%20proportion%20of%20UK%20adults,(from%2013%25).).

7. Gupta P, Khan A, Kumar A. Social media use by patients in health care: a scoping review. International Journal of Healthcare Management. 2022;15(2):121–131.

8. Lee JW, Bance ML. Hearing loss. Practical neurology. 2019;19(1):28–35.

9. Zimmer M. "But the data is already public": on the ethics of research in Facebook. Ethics and information technology. 2010;12:313–325.

10. Hussain Z, Sheikh Z, Tahir A, Dashtipour K, Gogate M, Sheikh A, et al. Artificial Intelligence–Enabled Social Media Analysis for Pharmacovigilance of COVID-19 Vaccinations in the United Kingdom: Observational Study. JMIR Public Health and Surveillance. 2022;8(5):e32543.

11. Timeline of UK coronavirus lockdowns, March 2020 to March 2021; 2021. `https://www.instituteforgovernment.org.uk/sites/default/files/timeline-lockdown-web.pdf`.

12. Waterloo SF, Baumgartner SE, Peter J, Valkenburg PM. Norms of online expressions of emotion: Comparing Facebook, Twitter, Instagram, and WhatsApp. New media & society. 2018;20(5):1813–1831.

13. Barak A, Sadovsky Y. Internet use and personal empowerment of hearing-impaired adolescents. Computers in human behavior. 2008;24(5):1802–1815.

14. Pilling D, Barrett P. Text communication preferences of deaf people in the United Kingdom. Journal of deaf studies and deaf education. 2008;13(1):92–103.

15. Saroj A, Pal S. Use of social media in crisis management: A survey. International Journal of Disaster Risk Reduction. 2020;48:101584.

figure 2 Click here to access/download;Figure;heatmap.png

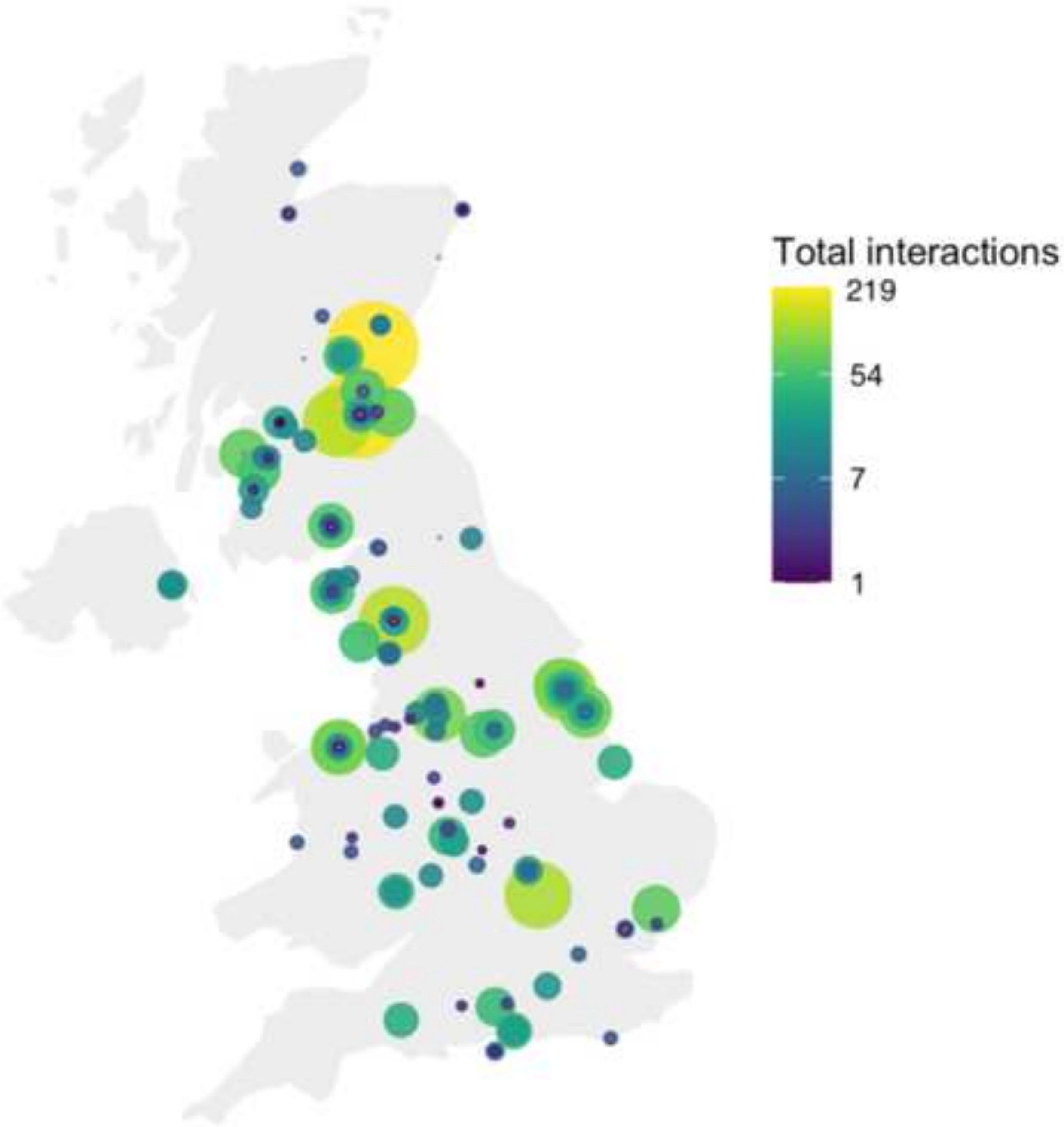

Figure 1

Click here to access/download;Figure;donut chart ss.png

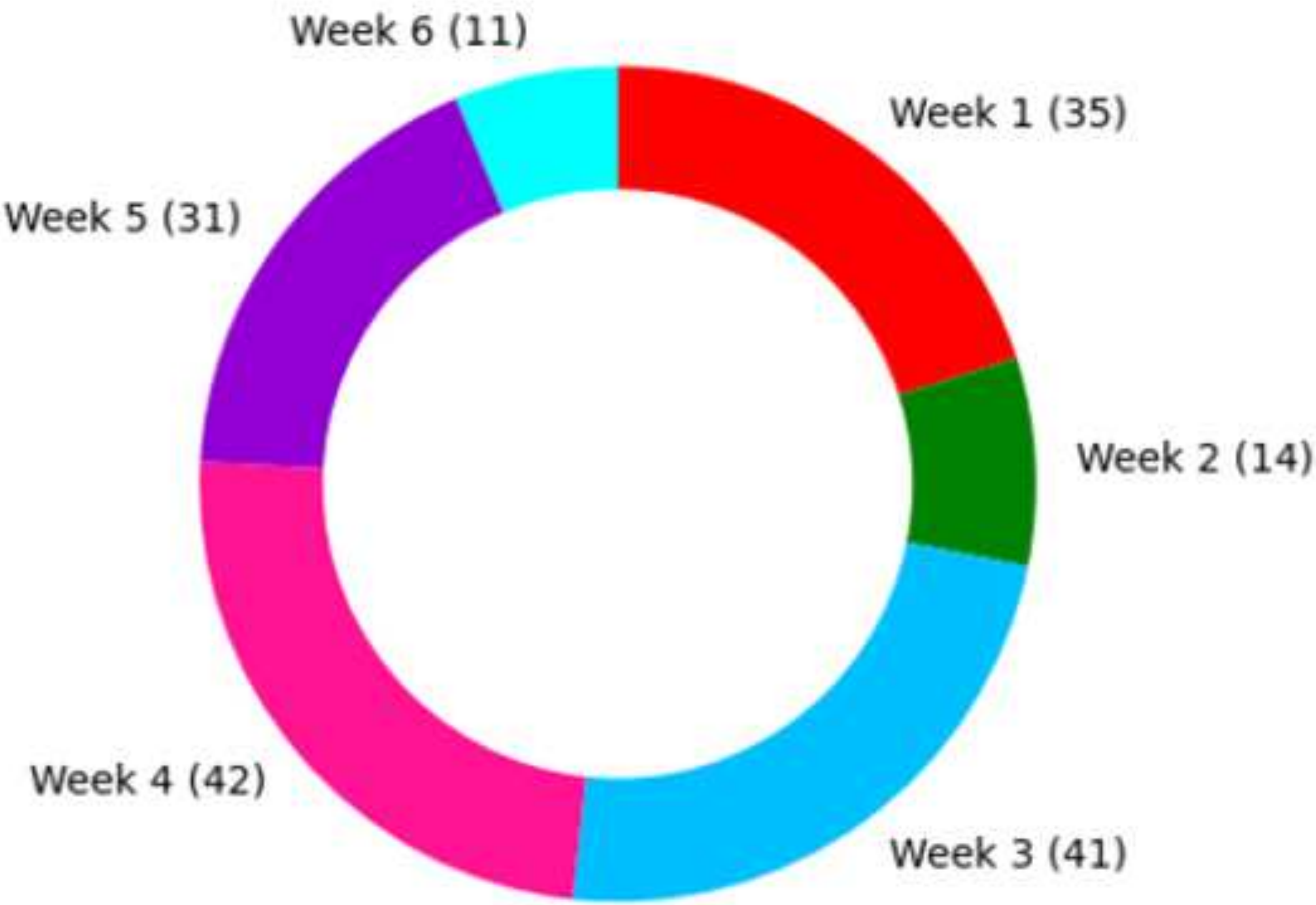

Reviewer comments re: **"Impact of the Covid-19 Pandemic on Audiology Service Delivery: Observational Study of the Role of Social Media in Patient Communication"**

See article comment bubbles for grammar and related comments.

The methodology employed in the study appears to be sound for the specific research question and objectives stated in the paper. The researchers aimed to investigate how NHS audiology departments in the UK utilized social media to communicate service changes during the Covid-19 pandemic. To achieve this goal, they collected data from Facebook and Twitter, focusing on a specific time frame (March 23 to April 30, 2020) that coincided with the initial announcement of the first UK lockdown.

The use of data extraction tools like Crowd tangle and Twitter API for collecting relevant posts aligns with the objective of analyzing social media communication. The researchers applied specific search terms to ensure comprehensive results and filtered the data manually to include only posts directly linked to NHS audiology service communication. Moreover, the study considered ethical considerations and data privacy laws, as the information was gathered from the public domain.

The geospatial mapping and interaction analysis were appropriate methods for analyzing the geographic distribution of posts and understanding user engagement with the content. The study also acknowledged its limitations, such as the inability to access private communications, which may have impacted the comprehensive understanding of all communication strategies.

Considering the research question and objectives, the methodology used allowed the researchers to gain insights into how NHS audiology departments used social media to communicate service changes during the pandemic. However, it is essential to recognize that the study's scope is specific to social media usage during a particular period and may not address all aspects of audiology service changes comprehensively.

To further enhance the study's depth and gain additional insights, the researchers could have considered conducting the following analyses:

Content Analysis: Instead of solely focusing on the number of interactions (likes, shares, comments), the researchers could have performed a content analysis of the posts. This analysis would involve categorizing the posts based on the type of information shared (e.g., changes in appointments, teleaudiology services, hearing aid maintenance), the tone of the messages (e.g., informative, reassuring, urgent), and the overall effectiveness of the communication.

Sentiment Analysis: By employing sentiment analysis, the researchers could assess the emotional tone of the social media posts. This analysis would help determine whether the messages conveyed by the audiology departments were perceived positively, negatively, or neutrally by the audience. Sentiment analysis can provide valuable insights into how well-received the service changes were among patients.

Comparison with Traditional Communication Channels: In addition to analyzing social media usage, the researchers could have compared the effectiveness of social media as a communication channel with more traditional methods like emails, SMS, or letters. By surveying patients who received information through different channels, the researchers could gauge the preferences and efficacy of various communication methods.

Longitudinal Analysis: The study focused on a specific six-week period during the initial announcement of the UK lockdown. A longitudinal analysis covering multiple time periods (e.g., before, during, and after the lockdown) could reveal how communication strategies evolved over time and how patients' responses changed as the pandemic progressed.

Qualitative Interviews or Surveys: To gain a more comprehensive understanding of patient perspectives and experiences, the researchers could have conducted qualitative interviews or surveys with patients who interacted with the audiology departments' social media posts. These interviews could provide valuable insights into patients' satisfaction, comprehension of information, and suggestions for improvement.

Comparison with Other Healthcare Specialties: The study focused on audiology service changes, but a broader analysis comparing social media communication practices across various healthcare specialties could highlight similarities, differences, and best practices for effective communication during a public health crisis.

Analysis of Responses to Misinformation: Since the study touched upon concerns about the credibility of information shared on social media, the researchers could have analyzed responses to misinformation related to audiology services during the pandemic. This analysis could help identify potential challenges in combating false or misleading content.

Cost-Benefit Analysis: A cost-benefit analysis of using social media as a communication channel could provide insights into the resource allocation for social media efforts compared to other communication methods. This analysis would be useful in determining the most cost-effective ways to reach and engage with patients.

By incorporating these additional analyses, the researchers could have gained a more comprehensive understanding of the role and impact of social media in engaging audiology consumers during the Covid-19 pandemic and beyond.
