# Supplementary material for: Impact of the Covid-19 pandemic on audiology service delivery: Observational study of the role of social media in patient communication
Source: PLoS One. 2024 Apr 25;19(4):e0288223. doi: 10.1371/journal.pone.0288223 (PMC11045075; doi:10.1371/journal.pone.0288223)
Supplement: Attachment — Submitted filename: Response to Reviewers.pdf [file pone.0288223.s002.pdf]

Response to Editor and Reviewer Comments

We would like to thank the Editor and Reviewers for their insightful comments on our study. We believe these have improved our study, and have provided point-by-point responses below.

Editor Comments:

1--Please spell out NHS the first time. I know in the UK it is well known but it took me a minute to get it.

Response: We have now spelled out as follows, “National Health Service (NHS)” , and updated this across the paper.

2–On line 22, please start a new paragraph

Response: We have amended accordingly.

3–line 32 has a change in topic so please be sure your paragraph is internally consistent

Response: We have amended this accordingly, and separated paragraphs.

4--Your introduction is extremely brief. COVID was not the first time that this type of service was online. I checked and there is a long history of moving towards this type of service. Please expand the introduction.

Response: We agree with respectable reviewer, we have added more information to the introduction clarifying that Teleaudiology had been looked into prior to Covid but still lacked infrastructure.

5--CrowdTangle (notice the Capital T) is not common knowledge. Therefore, you are going to need to explain that system in more detail. Also you have it spelled two different was":Crowd Tangle and Crowdtangle. Please use the correct name.

Response: Thank you for this comment, we have corrected CrowdTangle spelling within report, we have also detailed what CrowdTangle is capable of for a better understanding of the reader.

6--please define API

Response: Thanks for the reviewer for highlighting this, it has now been defined appropriately within the script, Application Programming interface (API), we have also detailed what the API is capable of doing for a better understanding for the reader.

7--It is not clear what parameters you are targeting. Please expand on exactly what are your parameters of interest.

Response: We agree with respectable reviewer, we have clarified the parameters further so that they are understandable to the reader clearly

8--Please separate the results and discussion

Response: Thank you for this, we have now separated the results and discussion section.

9--you need a better key to explain Figure 1. I still am not clear what the colors mean

Response: We have explained this to further help clarify what the different colours are illustrating.

10--Table 1 is redundant to the text so please delete it. Rule is either a table or in the text--one or the other

Response: Thank you, we have removed Table 1.

11--You need more interpretation of your results

Response: We agree with this and have expanded our results and discussion sections to provide more detail.

12--Table 2 needs to be expanded for clarity.

Response: We agree with this, and have explained the table more clearly in the results.

<u>Reviewer 1 Comments:</u>

1. To further enhance the study's depth and gain additional insights, the researchers could have considered conducting the following analyses:
   Content Analysis: Instead of solely focusing on the number of interactions (likes, shares, comments), the researchers could have performed a content analysis of the posts. This analysis would involve categorizing the posts based on the type of

information shared (e.g., changes in appointments, teleaudiology services, hearing aid maintenance), the tone of the messages (e.g., informative, reassuring, urgent), and the overall effectiveness of the communication.

Response: We agree with the reviewer that content analysis would have been useful for further insights, however on our initial reading/scanning of interactions and comments on Facebook, we found these to be largely limited to short replies and ‘reactions’ to posts. It would be useful for us to analyse other social media sources, e.g. hearing aid user forums, twitter etc, for content analysis and correlate them to our high-level findings. We have now made reference to this in our future work and limitations section. We have however carried out sentiment analysis on the available comments, as discussed in the next response.

2. Sentiment Analysis: By employing sentiment analysis, the researchers could assess the emotional tone of the social media posts. This analysis would help determine whether the messages conveyed by the audiology departments were perceived positively, negatively, or neutrally by the audience. Sentiment analysis can provide valuable insights into how well-received the service changes were among patients.

Response: Thank you for this insightful comment. We agree it would be useful to understand public sentiment towards these posts. As noted earlier, most of the patient engagement was limited to short comments or ‘reactions’ to posts with different emotions. However, we have extracted available comments and performed high level sentiment analysis on them , to gain some insight into public sentiment towards the service communications.

3. Comparison with Traditional Communication Channels: In addition to analyzing social media usage, the researchers could have compared the effectiveness of social media as a communication channel with more traditional methods like emails, SMS, or letters. By surveying patients who received information through different channels, the researchers could gauge the preferences and efficacy of various communication methods.

Response: This is an excellent suggestion, however we feel a larger experiment would be required where we obtain consent from patients enrolled to access their other communications. We have made reference to this in our future work.

4. Longitudinal Analysis: The study focused on a specific six-week period during the initial announcement of the UK lockdown. A longitudinal analysis covering multiple time periods (e.g., before, during, and after the lockdown) could reveal how communication strategies evolved over time and how patients' responses changed as the pandemic progressed.

Response: This is an excellent suggestion, however we feel a larger experiment would be required where we obtain consent from patients enrolled to access their other communications. We have made reference to this in our future work.

5. Qualitative Interviews or Surveys: To gain a more comprehensive understanding of patient perspectives and experiences, the researchers could have conducted qualitative interviews or surveys with patients who interacted with the audiology departments' social media posts. These interviews could provide valuable insights into patients' satisfaction, comprehension of information, and suggestions for improvement.

Response: This is an excellent suggestion, however we feel a larger experiment would be required where we obtain consent from patients enrolled to access their other communications. We have made reference to this in our future work.

6. Comparison with Other Healthcare Specialties: The study focused on audiology service changes, but a broader analysis comparing social media communication practices across various healthcare specialties could highlight similarities, differences, and best practices for effective communication during a public health crisis.

Response: This is an excellent suggestion, however we feel a larger experiment would be required where we obtain consent from patients enrolled to access their other communications. We have made reference to this in our future work.

7. Analysis of Responses to Misinformation: Since the study touched upon concerns about the credibility of information shared on social media, the researchers could have analyzed responses to misinformation related to audiology services during the pandemic. This analysis could help identify potential challenges in combating false or misleading content.

Response: This is an excellent suggestion, however we feel a larger experiment would be required where we obtain consent from patients enrolled to access their other communications. We have made reference to this in our future work.

8. Cost-Benefit Analysis: A cost-benefit analysis of using social media as a communication channel could provide insights into the resource allocation for social media efforts compared to other communication methods. This analysis would be useful in determining the most cost-effective ways to reach and engage with patients.

Response: This is another excellent suggestion, however we feel a larger experiment would be required where we obtain consent from patients enrolled to access their other communications. We have made reference to this in our future work.

Reviewer 2 comments:

1. p. 2, lines 32-33: You cite a study concluding that Facebook and Twitter are the most commonly used types of social media. Was any effort made to determine the types of social media most commonly used by the ages and other demographics of people who tend to use audiology services more regularly than others? Given that older adults are one of the largest groups seen by audiologists and that "presbycusis" was one of your search terms, I'm wondering whether your choices of Facebook and Twitter were based at all on older adult social media use patterns.

Response:Thank you for this comment from the reviewer. To further support the use of Facebook and Twitter within this study another study has been cited were the authors had found that the use of facebook and twitter was larger in the older population in comparison to other social platforms.

2. Also on the topic of populations that use audiology services: the search terms that you list seem primarily focused on adult audiology services. Were pediatric audiology services included in the social media posts that you analyzed? Or was this study primarily about adult audiology services?

Response: Thank you for the comment from the reviewer, all terminology used within the search terms would have been appropriate for both adult and children services except “Presbycusis” which refers to age related hearing loss.

3. p. 3, lines 94-95: Is there a reason to believe that only hearing aid users are interacting with these posts and therefore represented in the total number of interactions? The meaning of the sentence here isn't entirely clear to me. Are you trying to point out that not all hearing aid users are being reached by social media posts? Something else?

Response: Many thanks for the reviewer for this comment, we have clarified the sentence so that it is more understandable, “some of the interactions may not have been from directly from the hearing aid user but possibly a friend or family member who may be affected by this change of service. Interactions included likes, shares, comments, or reposts. However,

the reported interaction frequencies may underestimate the full reach, as some individuals may have viewed the posts without actively engaging through likes/comments.”

4. p. 4, lines 110-111: Why is YouTube coming up this far into the paper when Facebook and Twitter were the platforms used for collecting data for the study? And if 40% of participants reported using Facebook and YouTube in the study you cite at this point in the paper, is there a reason that you didn't use YouTube as a data source? I see that later in the paper, you mention YouTube as a future direction. Still, the mention of YouTube in the Results and Discussion section is somewhat confusing.

Response: Thank you to the reviewer for this comment. We have now ensured that the mention of specific social media platforms is now clearer and in a more appropriate order.
